# Synthesis and Biological Activity of Some 3-(4-(Substituted)-piperazin-1-yl)cinnolines

**DOI:** 10.3390/molecules17010227

**Published:** 2011-12-28

**Authors:** Eman D. Awad, Mustafa M. El-Abadelah, Suzan Matar, Malek A. Zihlif, Randa G. Naffa, Ehab Q. Al-Momani, Mohammad S. Mubarak

**Affiliations:** 1 Department of Chemistry, Faculty of Science, The University of Jordan, Amman 11942, Jordan; 2 Department of Biological Sciences, Faculty of Science, The University of Jordan, Amman 11942, Jordan; 3 Department of Pharmacology, Faculty of Medicine, The University of Jordan, Amman 11942, Jordan; 4 Laboratory of Molecular Biology Research, Faculty of Medicine, The University of Jordan, Amman 11942, Jordan; 5 Clinic of Nuclear Medicine, Ulm University, Ulm 89081, Germany

**Keywords:** hydrazonoyl chlorides, 3-(piperazin-1-yl)cinnolines, antitumor and antifungal activity

## Abstract

A new series of 6-substituted-4-methyl-3-(4-arylpiperazin-1-yl)cinnolines **8**–**10** were synthesized as potential antifungal agents via intramolecular cyclization of the respective 1-(2-arylhydrazono)-1-(4-arylpiperazin-1-yl)propan-2-ones **5**–**7**, mediated by polyphosphoric acid (PPA). The amidrazones themselves were synthesized via direct interaction of the appropriate hydrazonoyl chlorides **4a**–**d** with the corresponding *N*-substituted piperazine in the presence of triethylamine. The structures of the new prepared compounds were confirmed by elemental analyses, ^1^H-NMR, ^13^C-NMR, and ESI-HRMS spectral data. The antitumor, antibacterial, and antifungal activity of the newly synthesized compounds was evaluated.

## 1. Introduction

The benzo[*c*]pyridazine nucleus, better-known as cinnoline, and its derivatives have received considerable interest due to their wide range of pharmacological profiles, e.g., antibacterial [1], antitumor [2,3], antifungal [4] and anti-inflammatory [5] activities. Certain compounds of the cinnoline series have antithrombocytic [6] and antituberculosis [7] properties, and also exhibit anesthetizing [8], and sedative [9] activity, in addition to their use as agrochemicals [10].

**Figure 1:**
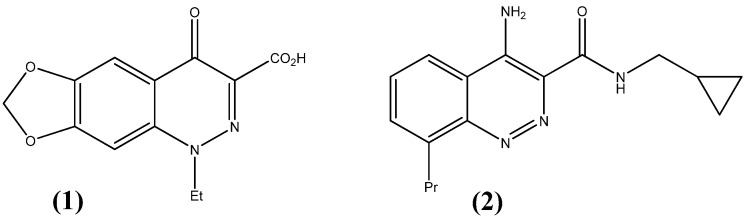
Cinoxacine (**1**) and ICI-D-7569 (**2**).

Significant commercial interest in the development of benzopyridazine derivatives, particularly pharmaceutical uses of pyridazines and cinnolines, is shown by the large number of patents filed in this area [11]. Their ring system is an isosteric relative to either quinoline or isoquinoline, therefore, in many cases the synthesized compounds were designed as analogs of previously obtained quinoline or isoquinoline derivatives; for example cinoxacin (**1**) is a cinnoline analogue of the quinoline antibacterials used for urinary tract infection [12] and ICI-D-7569 (**2**) is an anxiolytic agent [13] (Figure 1). Meanwhile, attention has been paid to the synthesis of heterocyclic compounds bearing a cinnoline moiety; an excellent review on the synthesis and characteristics of cinnolines has been published by Haider and colleagues [14].

**Scheme 1:**
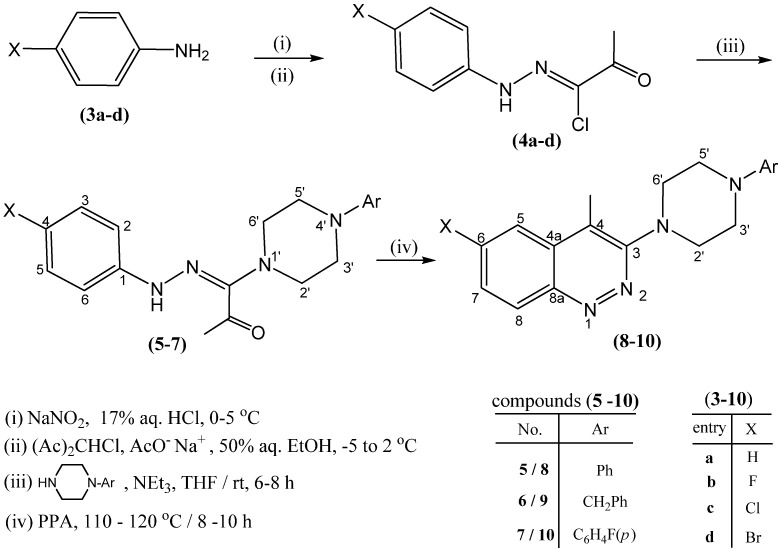
The synthetic route for compounds **8**–**10**.

In view of the interest in the activity spectrum and profile of cinnolines, and in continuation of our work on the synthesis of new compounds of pharmacological and biological interest [15,16,17], we describe herein the preparation and spectroscopic characterization of some new 3-(4-(substituted)-piperazin-1-yl)cinnolines (shown in Scheme 1), together with their antitumor, and antifungal activities.

## 2. Results and Discussion

### 2.1. Chemistry

The synthesis of 3-piperazinyl cinnolines **8**–**10** was carried out via interamolecular cyclization of the piperazinyl amidrazones **5**–**7** using PPA as a cyclizing agent as shown in Scheme 1. Syntheses of the respective amidrazones **5**–**7** in good yield were achieved according to a modified procedure [15] which involved treatment of the appropriate hydrazonyl chloride **4a**–**d** with *N*-substituted piperazine in the presence of triethylamine. Compounds **4a**–**d** were prepared by coupling of the respective arenediazonium salts with 3-chloro-2,4-pentanedione via the Japp-Klingemann reaction [18,19,20], according to reported procedures [21,22].

In the ^1^H-NMR (CDCl_3_) spectra of cinnoline derivatives, a singlet peak appears in the range *δ* 2.50–2.95 ppm corresponding to the methyl protons. The methylene protons of the piperazine moiety appear as two broad singlets or multiplet peaks in the range *δ* 3.32–3.48 ppm and *δ* 2.68–3.61 ppm. The aromatic protons signals resonate around *δ* 6.78–8.69 ppm. In the ^13^C-NMR spectra of compounds **8**–**10**, the methyl (CH_3_) carbon, resonates upfield between *δ* 12.6–17.7 ppm, which is indicative of the formation of cyclized product through acylation of the benzene ring; the methylene carbons of the piperazine moiety appear around *δ* 49.5–50.9 and 50.7–53.5 ppm, while the aromatic carbons resonate in the range *δ* 105.5–164.6 ppm.

### 2.2. Antibacterial and Antifungal Activity

#### 2.2.1. Compound Susceptibility Testing by Kirby Bauer Method

The newly synthesized compounds **8**–**10** were screened for their antibacterial activity against Gram negative (*Escherechia coli* ATCC 8739) and Gram-positive (*Staphylococcus aureus* ATCC 25923) microorganisms at 25 mg/mL. *In-vitro* antibacterial screening of the compounds showed that they were inactive against both organisms. In addition, these compounds were also inactive against *Candida glabirata* clinical neonatal isolates 1 and 2.

In addition, whereas compounds **8a**–**d** and **9a**–**d** showed no activity against *C. albicans* ATCC 10231and *C. glabirata* ATCC 15126, respectively, fairly good activity was found when tested against *C. albicans* clinical isolates (compounds **8a**–**d**, **9c**, **10b** and **10c**) with a percentage of inhibition zone of 40%–55% when compared to nystain. These results are shown in Table 1.

**Table 1:** Antifungal activity of compounds (**8**–**10**) at 25 mg/mL.

**Compound (25 µg/mL)**	**8a**	**8b**	**8c**	**8d**	**9a**	**9b**	**Nystatin**
*C. albicans* ATCC 10231	NA	NA	NA	NA	7 ± 0.2	7 ± 0.2	17 ± 1.5
*C. glabirata* ATCC 15126	8 ± 0.5	7 ± 0.1	7 ± 0.8	7 ± 0.5	NA	NA	20 ± 0.5
*C. albicans* Clinical isolate	7 ± 0.5	7 ± 0.1	7 ± 0.5	7 ± 0.1	8 ± 0.6	7 ± 0.2	15 ± 1
**Compound (25 µg/mL)**	**9c**	**9d**	**10a**	**10b**	**10c**	**10d**	**Nystain**
*C. albicans* ATCC 10231	NA	7 ± 0.2	8 ± 0.4	NA	NA	7 ± 0.5	17 ± 1.5
*C. glabirata* ATCC 15126	NA	NA	8 ± 0.5	8 ± 0.7	8 ± 0.4	9 ± 0.3	20 ± 0.5
*C. albicans* Clinical isolate	7 ± 0.6	7 ± 0.5	8 ± 0.3	7 ± 0.1	7 ± 0.1	8 ± 0.2	15 ± 1

The results are the mean ± SD (n = 3) in unit of mm. The well is 6 mm wide. NA: inactive at 25 mg/mL of the compound tested. Nystatin impregnated discs with 5 mm wide wells.

#### 2.2.2. Compound Susceptibility Testing by Microbroth Dilution Method

As has been mentioned earlier, compounds **8a**–**d** did not have any activity against *C. albicans* ATCC 10231 strain and *C. galibrata* clinical isolates 1 and 2; but they showed fungicidal rather than fungistatic activity in the range 0.2–3.0 mg/mL against *C. galibrata* ATCC 15126 strain. The minimum inhibitory concentration (MIC) of these compounds ranged from 0.3–5.0 mg/mL against *C. albicans* clinical isolates as displayed in Table 2. Results in Table 2 reveal that compounds **8a**, **8b** and **8c** are more effective, with no significant difference against *C. albicans* clinical isolate and *C. galibrata* ATCC 15126 strains when compared to **8d**, which has an MIC value of 3.0 mg/mL against the same strains.

**Table 2:** The MIC and MFC in mg/mL for compounds (**8a**–**d**) against *Candida* species.

Entry	8a		8b		8c		8d		Nystatin	
	MIC	MFC	MIC	MFC	MIC	MFC	MIC	MFC	MIC	MFC
*C. albicans* clinical isolate	0.4	0.9	0.3	0.6	0.4	0.9	3.0	5.0	0.008	0.03
*C.galibrata* ATCC 15126	0.4	0.4	0.2	0.2	0.4	0.4	3.0	3.0	0.003	0.02

NA: inactive, MFC: minimum fungicidal concentration.

The fungicidal concentration values (MFC) in Table 2 reveal that compounds **8a**–**d** displayed fungicidal activity against *C. galibrata* ATCC 15126 in the concentration range 0.2–3.0 mg/mL*.* On the other hand, the MFCs of the same compounds against *C. albicans* clinical isolate were in the 0.9–5.0 mg/mL range. We conclude that compounds **8a**–**d** MFC corresponds to about ¼–½ lesser concentration against *C. galibrata* ATCC 15126 when compared to *C. albicans* clinical isolate.

Shown in Table 3 is the antifungal activity of compounds **9a**–**d**. The results reveal that these compounds have no antifungal activity against *C. galibrata* clinical isolates (1 and 2), and *C. albicans* ATCC 10231; the results also show that **9a** and **9d** display fungicidal activity against *C. galibrata* ATCC 15126 strain. The results in Table 3 indicate that compounds **9a**, **9b**, and **9d**, exhibit moderate antifungal activity against *C. albicans* strains (ATCC 10231 and clinical isolate) and *C. galibrata* ATCC 15126 strain only, while **9c** is inactive.

**Table 3:** The MIC and MFC in mg/mL for compounds (**9a**–**d**) against *Candida* species.

Entry	9a		9b		9c		9d		Nystatin	
	MIC	MFC	MIC	MFC	MIC	MFC	MIC	MFC	MIC	MFC
*C .albicans* ATCC 10231	0.6	1.0	0.6	1.0	NA	NA	0.8	2.0	0.006	0.03
*C. albicans* clinical isolate	0.2	0.3	0.6	1.0	0.08	0.2	0.4	0.8	0.008	0.03
*C. galibrata* ATCC 15126	0.3	0.3	0.6	1.0	0.16	0.3	0.4	0.4	0.003	0.02

NA: inactive.

In conclusion, the prepared compounds included in this study have no antibacterial effect. In addition, the tested compounds have no activity against *C. galibrata* clinical isolates (1 and 2) but some antifungal activity against *C. albicans* clinical isolate. Some of the tested compounds such as **10c**, **10d** and **8a**–**d** have fungicidal activity rather than fungistatic effects. However, only **9a** had bactericidal activity against *E. coli* strain with MBC of 1.0 mg/mL.

These analyses emphasize the possible diversity in mechanisms that result in a phenotype of compounds resistance and selectivity amongst bacterial and fungal strains. These compounds should not be considered at this stage as potent therapeutic agents in mycosis especially when compared to nystatin. However selective compounds with antifungal activity could be potential agents in industrial mycology and microbiology especially if they prove to have low cytotoxicity in humans and animals. In addition; we emphasize the necessity for further work to modify the structures of the compounds to increase their activity firstly and secondly to decrease their cytotoxity in humans and animals.

### 2.3. Antitumor Activity

The antitumor activity of compounds **8**–**10** was characterized by conducting cell viability assays using the tetrazolium dye 3-(4,5-dimethylthiazol-2-yl)-2,5-diphenyltetrazolium bromide (MTT). Cultures of MCF-7 breast cancer cells were treated first at a concentration of 50 µg/mL and the results are shown in Table 4.

**Table 4:** Percentage cell survival of MCF-7 following 72 h exposure to 50 µg/mL of **8**–**10**.

Compound	8a	8b	8c	8d	9a	9b	9c	9d	10a	10b	10c	10d
MCF-7% survival	95	43	92	101	99	102	102	94	101	42	74	45
Standard deviation	±2.60	±3.53	±1.85	±1.10	±7.02	±3.21	±2.49	±0.71	±2.25	±1.48	±1.84	±2.31

Compounds **10b**, **10d**, and **8b** showed potential anti-MCF-7 activity and were able to reduce the viability after 72 h to less than 50% (Table 4). The anti-leukemic effect of these compounds was next tested against the K562 cell line, but none has shown any activity at ≤100 µg/mL. Furthermore, we determined the IC_50_ values for compounds **8b**, **10b** and **10d** on the MCF-7. Results, which are shown in Table 5, clearly reveal that compound **8b** was the most potent against MCF-7 cells, scoring an IC_50_ value of 5.56 μM. Compounds **10b** and **10d** have IC_50_ values of 11.79 and 8.57, respectively.

**Table 5:** Effect of compounds **8b**, **10b**, and **10d** on MCF-7.

Compound	8b	10b	10d	Doxorubicin
IC_50_ MCF-7 (μM)	5.56 ± 0.30	11.79 ± 2.05	8.57 ± 0.85	0.31 ± 0.01

## 3. Experimental

### 3.1. General

Melting points were recorded on SMP1 Stuart apparatus and are uncorrected. The ^1^H- and ^13^C-NMR spectra were recorded on a Bruker DPX-300 spectrometer in CDCl_3_ with TMS as an internal standard. The chemical shifts are reported in parts per million (ppm) expressed in *δ* units; coupling constant (*J*) values are given in Hertz (Hz). High resolution mass spectra (HRMS) were acquired using electrospray ionization (ESI) technique on a Bruker APEX-4 instrument. The samples were dissolved in CDCl_3_, diluted in spray solution (methanol/water 1:1 v/v + 0.1% formic acid) and infused using a syringe pump with a flow rate of 2 µL/min. External calibration was conducted using arginine cluster in a mass range *m/z* 175–871. Elemental analyses were performed on a Euro Vector Elemental Analyzer (EA 3000 A). The following chemicals were used as received without further purification: Substituted anilines and polyphosphoric acid (Fluka), 3-chloro-2,4-pentanedione, 1-(4-fluorophenyl)-piperazine, 1-phenylpiperazine, 1-benzylpiperazine (Acros). The reactions were monitored by thin layer chromatography (TLC), carried out on silica gel plates (60 F-254, Scharlau). Plates were visualized under UV light (where appropriate). Preparative thick layer chromatography was performed on 0.5 mm silica gel glass plates (60 F-254, Scharlau).

### 3.2. General Procedure for the Synthesis of Substituted Piperazin-1-yl amidrazones ***5**–**7***

To a stirred solution of 1-chloro-1-(4-subsitituted) phenylhydrazono)propan-2-one **4a**–**d** (10 mmol) and triethylamine (3 mL) in THF (10 mL) was added the appropriate piperazine (25 mmol), and the resulting mixture was stirred at room temperature for 6–8 h. The reaction mixture was then diluted with water (60 mL) and extracted with diethyl ether (3 × 50 mL). The combined organic extracts were dried over anhydrous sodium sulfate, and the solvent was evaporated under reduced pressure. The obtained residue was purified by recrystalization from ethanol.

*1-(2-(4-Fluorophenyl)hydrazono)-1-(4-phenylpiperazin-1-yl)propan-2-one* (**5b**). Yield: 76%; mp = 123–125 °C. ^1^H-NMR (CDCl_3_) *δ*: 2.43 (3H, s, CH_3_), 3.24 (4H, m, H_2_-3'+H_2_-5'), 3.26 (4H, m, H_2_-2'+H_2_-6'), 6.87–7.32 (9H, m, Ar), 9.15 (1H, s, N–H). ^13^C-NMR (CDCl_3_) *δ*: 25.8 (CH_3_), 48.1 (C-2'/C-6'), 50.3 (C-3'/C-5'), 115.4 (d, ^3^*J*_C-F_ = 7.5 Hz, C–H), 116.2 (d, ^2^*J*_C-F_ = 22.5 Hz, C–H), 116.4 (C–H), 120.3 (C–H), 129.3 (C–H), 139.0 (C), 143.3 (C), 151.5 (C), 158.6 (d, ^1^*J*_C-F_ = 240.0 Hz, C), 195.1 (C=O). HRMS (ESI) *m/z*: 341.16797 (Calcd for C_19_H_22_FN_4_O [M+H]^+^: 341.16994). Anal. Calcd for C_19_H_21_FN_4_O: C, 67.04; H, 6.22; N, 16.46. Found: C, 66.88; H, 6.19; N, 16.33.

*1-(4-Benzylpiperazin-1-yl)-1-(2-phenylhydrazono)propan-2-one* (**6a**). Yield: 76%; mp = 102–105 °C. ^1^H-NMR (CDCl_3_) *δ*: 2.41 (3H, s, CH_3_), 2.54 (4H, m, H_2_-3'+H_2_-5'), 3.06 (4H, m, H_2_-2'+H_2_-6'), 3.57 (2H, s, C*H*_2_Ph), 6.97 (1H, t, *J =* 7.3, H-4), 7.17–7.37 (10H, m, Ar), 9.13 (1H, s, N–H). ^13^C-NMR (CDCl_3_) *δ*: 25.7 (CH_3_), 47.8 (C-2'/C-6'), 53.8 (C-3'/C-5'), 63.2 (*C*H_2_Ph), 114.1 (C–H), 122.0 (C–H), 127.1 (C–H), 128.3 (C–H), 129.1 (C–H), 129.4 (C–H), 138.0 (C), 142.5 (C), 143.5 (C), 195.0 (C=O). HRMS (ESI) *m/z*: 337.20029 (Calcd for C_20_H_25_N_4_O [M+H]^+^: 337.19501). Anal*.* Calcd for C_20_H_24_N_4_O: C, 71.40; H, 7.19; N, 16.65. Found: C, 71.48; H, 7.17; N, 16.57.

*1-(4-Benzylpiperazin-1-yl)-1-(2-(4-fluorophenyl)hydrazono)propan-2-one* (**6b**). Yield: 76%; mp = 121–124 °C. ^1^H-NMR (CDCl_3_) *δ*: 2.39 (3H, s, CH_3_), 2.53 (4H, m, H_2_-3'+H_2_-5'), 3.06 (4H, m, H_2_-2'+H_2_-6'), 3.57 (2H, s, C*H*_2_Ph), 6.99–7.04 (2H, m, C_6_H_4_F), 7.11–7.14 (2H, m, C_6_H_4_F) 7.25–7.37 (5H, m, Ar), 9.08 (1H, s, N–H). ^13^C-NMR (CDCl_3_) *δ*: 25.7 (CH_3_), 47.8 (C-2'/C-6'), 53.8 (C-3'/C-5'), 63.2 (*C*H_2_Ph), 115.1 (d, ^3^*J*_C-F_ = 7.5 Hz, C–H), 116.1 (d, ^2^*J*_C-F_ = 22.5 Hz, C–H), 127.1 (C–H), 128.3 (C–H), 129.1 (C–H), 138.0 (C), 138.9 (C), 143.5 (C), 158.3 (d, ^1^*J*_C-F_ = 240.0 Hz, C), 194.8 (C=O). HRMS (ESI) *m/z*: 355.18287 (Calcd for C_20_H_24_FN_4_O [M+H]^+^: 355.18559). Anal. Calcd for C_20_H_23_FN_4_O: C, 67.78; H, 6.54; N, 15.81. Found: C, 67.87; H, 6.50; N, 15.72.

*1-(4-Benzylpiperazin-1-yl)-1-(2-(4-chlorophenyl)hydrazono)propan-2-one* (**6c**). Yield: 63%; mp = 115–117 °C. ^1^H-NMR (CDCl_3_) *δ*: 2.41 (3H, s, CH_3_), 2.54 (4H, m, H_2_-3'+H_2_-5'), 3.06 (4H, m, H_2_-2'+H_2_-6'), 3.57 (2H, s, C*H*_2_Ph), 6.92–6.95 (1H, m, Ar), 7.01–7.04 (1H, m, Ar), 7.20–7.37 (m, 7H, Ar), 9.08 (1H, s, N–H). ^13^C-NMR (CDCl_3_) *δ*: 25.8 (CH_3_), 47.8 (C-2'/C-6'), 53.8 (C-3'/C-5'), 63.1 (*C*H_2_Ph), 114.2 (C–H), 127.1 (C–H), 128.3 (C–H), 129.0 (C–H), 130.4 (C–H), 135.2 (C), 138.0 (C), 143.8 (C), 144.2 (C), 195.0 (C=O). HRMS (ESI) *m/z*: 371.15332 (Calcd for C_20_H_24_ClN_4_O [M+H]^+^: 371.15604).Anal. Calcd for C_20_H_23_ClN_4_O: C, 64.77; H, 6.25; N, 15.11. Found: C, 64.56; H, 6.27; N, 15.06.

*1-(4-Benzylpiperazin-1-yl)-1-(2-(4-bromophenyl)hydrazono)propan-2-one* (**6d**). Yield: 96%; mp = 106–108 °C. ^1^H-NMR (CDCl_3_) *δ*: 2.39 (3H, s, CH_3_), 2.53 (4H, m, H_2_-3'+H_2_-5'), 3.04 (4H, m, H_2_-2'+H_2_-6'), 3.56 (2H, s, C*H*_2_Ph), 7.06 (2H, d, *J =* 8.7, Ar), 7.40 (2H, d, *J =* 8.7, Ar), 7.25–7.34 (5H, m, Ar), 9.08 (1H, s, N–H). ^13^C-NMR (CDCl_3_) *δ*: 25.7 (CH_3_), 47.8 (C-2'/C-6'), 53.7 (C-3'/C-5'), 63.1 (*C*H_2_Ph), 115.6 (C–H), 127.1(C–H), 128.2 (C–H), 129.1(C–H), 132.2 (C–H), 138.0 (C), 141.3 (C), 141.7 (C), 143.9 (C), 194.9 (C=O). HRMS (ESI) *m/z*: 415.10680 (Calcd for C_20_H_24_BrN_4_O [M+H]^+^: 415.10552). Anal. Calcd for C_20_H_23_BrN_4_O: C, 57.84; H, 5.58; N, 13.49. Found: C, 57.72; H, 5.60; N, 13.36.

*1-(2-(4-Fluorophenyl)hydrazono)-1-(4-(4-fluorophenyl)piperazin-1-yl)propan-2-one* (**7b**). Yield: 72%; mp = 157–159 °C. ^1^H-NMR (CDCl_3_) *δ*: 2.42 (3H, s, CH_3_), 3.20 (8H, br m, H_2_-3'+H_2_-5'/H_2_-2'+H_2_-6'), 6.88–7.17 (8H, m, Ar), 9.13 (1H, s, N–H). ^13^C-NMR (CDCl_3_) *δ*: 25.7 (CH_3_), 48.0 (C-2'/C-6'), 51.1 (C-3'/C-5'), 115.2 (d, ^3^*J*_C-F_ = 7.5 Hz, C–H), 115.6 (d, ^2^*J*_C-F_ = 22.5 Hz, C–H), 116.1 (d, ^2^*J*_C-F_ = 22.5 Hz, C–H), 118.2 (d, ^3^*J*_C-F_ = 7.5 Hz, C–H), 138.8 (C), 143.1 (C), 148.0 (C), 157.3 (d, ^1^*J*_C-F_ = 233.0 Hz, C), 158.4 (d, ^1^*J*_C-F_ = 240.0 Hz, C), 194.90 (C=O). HRMS (ESI) *m/z*: 359.15970 (Calcd for C_19_H_21_F_2_N_4_O [M+H]^+^: 359.16052). Anal. Calcd for C_19_H_20_F_2_N_4_O: C, 63.68; H, 5.62; N, 15.63. Found: C, 63.58; H, 5.66; N, 15.70.

### 3.3. General Procedure for the Synthesis of 4-methyl-3-[(4-substituted)piperazin-1-yl]cinnolines ***8**–**10***

A solution of the appropriate piperazinyl amidrazone **5**–**7** (1.5 mmol) in PPA (5.0 g) was stirred at 110–120 °C for 8–10 h. The reaction mixture was then cooled to room temperature, treated with crushed ice (10 g), and neutralized with 10% aqueous ammonium hydroxide. The reaction mixture was then extracted with ethyl acetate (3 × 50 mL) and the combined organic extracts were evaporated under reduced pressure to afford crude residue of the respective title compound which was recrystallized from ethanol.

*4-Methyl-3-(4-phenylpiperazin-1-yl)cinnoline* (**8a**). Yield: 75%; mp = 164–166 °C. ^1^H-NMR (CDCl_3_) *δ*: 2.64 (3H, s, CH_3_), 3.42 (4H, m, H_2_-3'+H_2_-5'), 3.56 (4H, m, H_2_-2'+H_2_-6'), 6.89 (1H, t, *J* = 7.2 Hz, Ar), 7.02 (2H, d, *J =* 8.0 Hz, Ar), 7.30 (2H, t, *J =* 8.0, Ar), 7.62–7.65 (2H, m, Ar), 7.89–7.92 (1H, m, Ar), 8.36–8.39 (1H, m, Ar). ^13^C-NMR (CDCl_3_) *δ*: 12.6 (*C*H_3_), 49.5 (C-2'/C-6'), 50.9 (C-3'/C-5'), 116.2 (C–H), 119.1 (C–H), 122.2 (C), 122.8 (C–H), 127.9 (C–H), 128.2 (C), 129.1 (C–H), 130.1 (C–H), 130.3 (C–H), 148.0 (C), 151.4 (C), 159.2 (C). HRMS (ESI) *m/z*: 305.17107 (Calcd for C_19_H_21_N_4_ [M+H]^+^: 305.16880). Anal. Calcd for C_19_H_20_N_4_: C, 74.97; H, 6.62; N, 18.41. Found: C, 74.88; H, 6.57; N, 18.22. 

*6-Fluoro-4-methyl-3-(4-phenylpiperazin-1-yl)cinnoline* (**8b**). Yield: 78%; mp = 196–198 °C. ^1^H-NMR (CDCl_3_) *δ*: 2.58 (3H, s, CH_3_), 3.43 (4H, m, H_2_-3'+H_2_-5'), 3.57 (4H, m, H_2_-2'+H_2_-6'), 6.89 (1H, t, *J* = 7.2 Hz, Ar), 7.02 (2H, d, *J* = 8.1 Hz, Ar), 7.28–7.33 (2H, m, Ar), 7.37–7.47 (2H, m, Ar), 8.37–8.43 (1H, m, Ar). ^13^C-NMR (CDCl_3_) *δ*: 13.0 (CH_3_), 49.6 (C-2'/C-6'), 50.9 (C-3'/C-5'), 105.9 (d, ^2^*J*_C-F_ = 22.5 Hz, C–H), 116.3 (C–H), 119.3 (d, ^2^*J*_C-F_ = 26.0 Hz, C–H), 120.0 (C–H), 121.2 (C), 129.3 (C–H), 129.8 (C), 133.7 (d, ^3^*J*_C-F_ = 11.0 Hz, C–H), 145.9 (C), 151.5 (C), 159.2 (C), 162.9 (d, ^1^*J*_C-F_ = 255.0 Hz, C). HRMS (ESI) *m/z*: 323.16065 (Calcd for C_19_H_20_FN_4_ [M+H]^+^: 323.15937). Anal. Calcd for C_19_H_19_FN_4_: C, 70.79; H, 5.94; N, 17.38. Found: C, 70.63; H, 6.01; N, 17.27.

*6-Chloro-4-methyl-3-(4-*phenylpiperazin*-1-yl)cinnoline* (**8c**). Yield: 43%; mp = 156–160 °C. ^1^H-NMR (CDCl_3_) *δ*: 2.95 (3H, s, CH_3_), 3.42 (4H, m, H_2_-3'+H_2_-5'), 3.57 (4H, m, H_2_-2'+H_2_-6'), 6.78–6.97 (1H, m, Ar), 7.02 (2H, d, *J =* 7.9, Ar), 7.26–7.33 (2H, m, Ar), 7.46–7.58 (1H, m, Ar), 7.67 (1H, d, *J =* 7.2, Ar), 8.30–8.36 (1H, m, H-8). ^13^C-NMR (CDCl_3_) *δ*: 17.7 (CH_3_), 49.5 (C-2'/C-6'), 50.7 (C-3'/C-5'), 116.2 (C–H), 119.9 (C–H), 124.6 (C), 127.3 (C–H), 128.5 (C), 129.1 (C–H), 130.2 (C–H), 131.5 (C), 132.5 (C–H), 149.1 (C), 151.3 (C), 160.7 (C). HRMS (ESI) *m/z*: 361.11905 (Calcd for C_19_H_19_ClN_4_Na [M+Na]^+^: 361.11959). Anal. Calcd for C_19_H_19_ClN_4_: C, 67.35; H, 5.65; N, 16.54. Found: C, 67.39; H, 5.54; N, 16.43.

*6-Bromo-4-methyl-3-(4-phenylpiperazin-1-yl)cinnoline* (**8d**). Yield: 20%; mp = 194–196 °C. ^1^H-NMR (CDCl_3_) *δ*: 2.59 (3H, s, CH_3_), 3.43 (4H, m, H_2_-3'+H_2_-5'), 3.56 (4H, m, H_2_-2'+H_2_-6'), 6.89 (1H, t, *J* = 7.2, Ar), 7.02 (2H, d, *J* = 8.0, Ar), 7.25–7.33 (2H, m, Ar), 7.69 (1H, d, *J* = 9.0, Ar), 8.10 (1H, s, Ar), 8.24 (1H, d, *J* = 9.0, Ar). ^13^C-NMR (CDCl_3_) *δ*: 12.8 (CH_3_), 49.5 (C-2'/C-6'), 50.9 (C-3'/C-5'), 116.2 (C–H), 120.0 (C–H), 120.5 (C), 125.3 (C–H), 125.6 (C–H), 129.2 (C–H), 131.6 (C–H), 132.0 (C–H), 132.7 (C), 146.4 (C), 151.4 (C), 159.5 (C). HRMS (ESI) *m/z*: 405.06853 (Calcd for C_19_H_19_BrN_4_Na [M+Na]^+^: 405.06908). Anal. Calcd for C_19_H_19_BrN_4_: C, 59.54; H, 5.00; N, 14.62. Found: C, 59.38; H, 4.93; N, 14.71.

*3-(4-Benzylpiperazin-1-yl)-4-methylcinnoline* (**9a**). Yield: 30%; mp = 162–164 °C. ^1^H-NMR (CDCl_3_) *δ*: 2.58 (3H, s, CH_3_), 2.71 (4H, m, H_2_-3'+H_2_-5'), 3.42 (4H, m, H_2_-2'+H_2_-6'), 3.63 (2H, s, CH_2_Ph), 7.24–7.40 (5H, m, Ar), 7.58–7.64 (2H, m, Ar), 7.84–7.89 (1H, m, Ar), 8.32–8.37 (1H, m, Ar). ^13^C-NMR (CDCl_3_) *δ*: 12.8 (CH_3_), 50.9 (C-2'/C-6'), 53.5 (C-3'/C-5'), 63.3 (*C*H_2_Ph), 121.8 (C), 123.0 (C–H), 127.2 (C–H), 127.8 (C–H), 128.4 (C–H), 129.3 (C–H), 130.3 (C–H), 132.9 (C), 138.1 (C), 148.0 (C), 159.5 (C). HRMS (ESI) *m/z*: 319.18272 (Calcd for C_20_H_23_N_4_ [M+H]^+^: 319.18445). Anal. Calcd for C_20_H_22_N_4_: C, 75.44; H, 6.96; N, 17.60. Found: C, 75.23; H, 6.91; N, 17.52.

*3-(4-Benzylpiperazin-1-yl)-6-fluoro-4-methylcinnoline* (**9b**). Yield: 20%; mp = 113–117 °C. ^1^H-NMR (CDCl_3_) *δ*: 2.50 (3H, s, CH_3_), 2.70 (4H, m, H_2_-3'+H_2_-5'), 3.42 (4H, m, H_2_-2'+H_2_-6'), 3.62 (2H, s, CH_2_Ph), 7.25–7.37 (7H, m, Ar), 8.33–8.38 (1H, m, Ar). ^13^C-NMR (CDCl_3_) *δ*: 13.0 (CH_3_), 50.6 (C-2'/C-6'), 53.3 (C-3'/C-5'), 63.2 (*C*H_2_Ph), 105.7 (d, ^2^*J*_C-F_ = 22.5 Hz, C–H), 118.9 (d, ^2^*J*_C-F_ = 26.0 Hz, C–H), 120.9 (C), 127.2 (C–H), 128.3 (C–H), 129.2 (C–H), 129.8 (C), 133.5 (d, ^3^*J*_C-F_ = 7.5 Hz, C–H), 137.9 (C), 145.7 (C), 159.3 (C), 162.7 (d, ^1^*J*_C-F_ = 255.0 Hz, C). HRMS (ESI) *m/z*: 337.17230 (Calcd for C_20_H_22_FN_4_ [M+H]^+^: 337.17502). Anal. Calcd for C_20_H_21_FN_4_: C, 71.41; H, 6.29; N, 16.65. Found: C, 71.38; H, 6.27; N, 16.54.

*3-(4-Benzylpiperazin-1-yl)-6-chloro-4-methylcinnoline* (**9c**). Yield: 60%; mp = 150–152 °C. ^1^H-NMR (CDCl_3_) *δ*: 2.53 (3H, s, CH_3_), 2.69 (4H, m, H_2_-3'+H_2_-5'), 3.43 (4H, m, H_2_-2'+H_2_-6'), 3.62 (2H, s, CH_2_Ph), 7.25–7.39 (5H, m, Ar), 7.51 (1H, dd, *J =* 1.9, 9.2, Ar), 7.84 (1H, d, *J* = 1.9, Ar), 8.28 (2H, d, *J* = 9.0, Ar). ^13^C-NMR (CDCl_3_) *δ*: 12.9 (CH_3_), 50.7 (C-2'/C-6'), 53.3 (C-3'/C-5'), 63.2 (*C*H_2_Ph), 120.3 (C), 121.7 (C–H), 127.1 (C–H), 128.3 (C–H), 128.9 (C–H), 129.0 (C), 129.2 (C–H), 132.0 (C–H), 136.6 (C), 138.0 (C), 146.1 (C), 159.6 (C). HRMS (ESI) *m/z*: 353.14475 (Calcd for C_20_H_22_ClN_4_ [M+H]^+^: 353.14547). Anal. Calcd for C_20_H_21_ClN_4_: C, 68.08; H, 6.00; N, 15.88. Found: C, 67.95; H, 5.87; N, 15.73.

*3-(4-Benzylpiperazin-1-yl)-6-bromo-4-methylcinnoline* (**9d**). Yield: 45%; mp = 153–155 °C. ^1^H-NMR (CDCl_3_) *δ*: 2.52 (3H, s, CH_3_), 2.70 (4H, m, H_2_-3'+H_2_-5'), 3.42 (4H, m, H_2_-2'+H_2_-6'), 3.62 (2H, s, CH_2_Ph), 7.24–7.39 (5H, m, Ar), 7.64 (1H, dd, *J* = 1.9, 9.0, Ar), 8.03 (1H, d, *J* = 1.9, Ar), 8.20 (1H, d, *J* = 9.0, Ar). ^13^C-NMR (CDCl_3_) *δ*: 13.0 (CH_3_), 50.8 (C-2'/C-6'), 53.4 (C-3'/C-5'), 63.3 (*C*H_2_Ph), 120.2 (C), 125.3 (C–H), 125.5 (C), 127.2 (C–H), 128.4 (C–H), 129.3 (C–H), 129.5 (C), 131.4 (C–H), 132.0 (C–H), 138.0 (C), 146.3 (C), 159.7 (C). HRMS (ESI) *m/z*: 419.08418 (Calcd for C_20_H_21_BrN_4_Na [M+Na]^+^: 419.08473). Anal. Calcd for C_20_H_21_BrN_4_: C, 60.46; H, 5.33; N, 14.10. Found: C, 60.29; H, 5.27; N, 13.99.

*3-(4-(4-Fluorophenyl)piperazin-1-yl)-4-methylcinnoline* (**10a**). Yield: 35%; mp = 183–185 °C. ^1^H-NMR (CDCl_3_) *δ*: 2.64 (3H, s, CH_3_), 3.35 (4H, m, H_2_-3'+H_2_-5'), 3.56 (4H, m, H_2_-2'+H_2_-6'), 6.95–7.03 (3H, m, Ar), 7.63–7.66 (2H, m, Ar), 7.89–7.92 (1H, m, Ar), 8.36–8.40 (1H, m, Ar). ^13^C-NMR (CDCl_3_) *δ*: 12.6 (CH_3_), 50.5, (C-2'/C-6'), 51.0 (C-3'/C-5'), 115.6 (d, ^2^*J*_C-F_ = 22.5 Hz, C–H), 118.0 (d, ^3^*J*_C-F_ = 7.5 Hz, C–H), 122.1 (C), 122.9 (C–H), 127.9 (C–H), 128.2 (C), 130.2 (C–H), 130.3 (C–H), 148.0 (C), 148.1 (C), 157.3 (d, ^1^*J*_C-F_ = 235.0 Hz, C), 159.1 (C). HRMS (ESI) *m/z*: 323.16065 (Calcd for C_19_H_20_FN_4_ [M+H]^+^: 323.15937). Anal. Calcd for C_19_H_19_FN_4_: C, 70.79; H, 5.94; N, 17.38. Found: C, 70.71; H, 5.89; N, 17.35.

*6-Fluoro-3-(4-(4-fluorophenyl)piperazin-1-yl)-4-methylcinnoline* (**10b**). Yield: 25%; mp = 173–176 °C. ^1^H-NMR (CDCl_3_) *δ*: 2.57 (3H, s, CH_3_), 3.34 (4H, m, H_2_-3'+H_2_-5'), 3.57 (4H, m, H_2_-2'+H_2_-6'), 6.93–7.03 (4H, m, Ar), 7.37–7.46 (2H, m, Ar), 8.40 (1H, dd, *J =* 5.7, 9.2, Ar). ^13^C-NMR (CDCl_3_) *δ*: 13.1 (CH_3_), 50.6 (C-2'/C-6'), 50.9 (C-3'/C-5'), 105.7 (d, ^2^*J*_C-F_ = 22.5 Hz, C–H), 115.5 (d, ^2^*J*_C-F_ = 22.5 Hz, C–H), 118.1 (d, ^3^*J*_C-F_ = 7.5 Hz, C–H), 119.4 (d, ^2^*J*_C-F_ = 26.0 Hz, C–H), 121.4 (C), 133.6 (d, ^3^*J*_C-F_ = 7.5 Hz, C–H), 145.9 (C), 148.1 (C), 153.7 (C), 157.4 (d, ^1^*J*_C-F_ = 240.0 Hz, C), 159.2 (C), 162.0 (d, ^1^*J*_C-F_ = 248.0 Hz, C). HRMS (ESI) *m/z*: 341.15123 (Calcd for C_19_H_19_F_2_N_4_ [M+H]^+^: 341.14995). Anal. Calcd for C_19_H_18_F_2_N_4_: C, 67.05; H, 5.33; N, 16.46. Found: C, 66.98; H, 5.38; N, 16.38.

*6-Chloro-3-(4-(4-fluorophenyl)piperazin-1-yl)-4-methylcinnoline* (**10c**). Yield: 75%; mp = 156–158 °C. ^1^H-NMR (CDCl3) *δ*: 2.59 (3H, s, CH_3_), 3.35 (4H, m, H_2_-3'+H_2_-5'), 3.56 (4H, m, H_2_-2'+H_2_-6'), 6.91–7.03 (4H, m, Ar), 7.69 (1H, d, *J =* 9.0, Ar), 8.08 (1H, s, Ar), 8.69 (1H, d, *J =* 9.1, Ar). ^13^C-NMR (CDCl_3_) *δ*: 12.9 (CH_3_), 50.6 (C-2'/C-6'), 50.9 (C-3'/C-5'), 115.6 (d, ^2^*J*_C-F_ = 21.0 Hz, C–H), 118.1 (d, ^3^*J*_C-F_ = 7.5 Hz, C–H), 120.3 (C), 121.8 (C–H), 129.0 (C), 129.4 (C–H), 131.8 (C–H), 138.1 (C), 146.1 (C), 147.5 (C), 157.4 (d, ^1^*J*_C-F_ = 235.0 Hz, C), 159.8 (C). HRMS (ESI) *m/z*: 357.12010 (Calcd for C_19_H_19_ClFN_4_ [M+H]^+^: 357.12040). Anal. Calcd for C_19_H_18_ClFN_4_: C, 63.95; H, 5.08; N, 15.70. Found: C, 63.82; H, 5.02; N, 15.53.

*6-Bromo-3-(4-(4-fluorophenyl)piperazin-1-yl)-4-methylcinnoline* (**10d**). Yield: 62%; mp = 208–210 °C. ^1^H-NMR (CDCl_3_) *δ*: 2.57 (3H, s, CH_3_), 3.34 (4H, m, H_2_-3'+H_2_-5'), 3.56 (4H, m, H_2_-2'+H_2_-6'), 6.92–7.02 (4H, m, Ar), 7.68 (1H, dd, *J =* 1.9, 9.0, Ar), 8.06 (1H, d, *J =* 1.8, Ar), 8.23 (1H, d, *J* = 9.0, Ar). ^13^C-NMR (CDCl_3_) *δ*: 12.8 (CH_3_), 50.5 (C-2'/C-6'), 50.8 (C-3'/C-5'), 115.6 (d, ^2^*J*_C-F_ = 22.5 Hz, C–H), 118.0 (d, ^3^*J*_C-F_ = 7.5 Hz, C–H), 120.5 (C), 125.2 (C–H), 125.6 (C), 129.4 (C), 131.6 (C–H), 131.9 (C–H), 146.4 (C), 148.0 (C), 157.3 (d, ^1^*J*_C-F_ = 240.0 Hz, C), 159.4 (C). HRMS (ESI) *m/z*: 401.07116 (Calcd for C_19_H_19_BrFN_4_ [M+H]^+^: 401.06989). Anal. Calcd for C_19_H_18_BrFN_4_: C, 56.87; H, 4.52; N, 13.96. Found: C, 56.80; H, 4.54; N, 13.85.

### 3.4. Biological Activity Test Procedures

#### 3.4.1. *Candida* Cultures

Compounds **8**–**10** were tested for their activity against *Candida* (fungi or yeast) strains using laboratory controls from American Type Culture Collection (ATCC) (Rockville, MD, USA) and clinical isolates which were a gift from Basem Jaber (The University of Jordan, Department of Biological Sciences): *Candida glabirata* ATCC 15126, *Candida albicans* clinical isolate (urinary tract infection), *Candida glabirata* clinical isolate 1 and 2 (neonate infections). Candida strains were cultured overnight at 37 °C in Sabouraud Dextrose broth.

#### 3.4.2. Compound Susceptibility Testing Disk Diffusion Method/(Kirby Bauer method)

The synthetic compounds **8**–**10** were tested *in vitro* for their antibacterial activity against Gram positive *S. aureus* ATCC 25923 and Gram negative *E. coli* ATCC 8739, and *Candida* at 25 mg/mL by modified Kirby-Bauer agar diffusion method [23,24].

The National Committee for Clinical Laboratory Standards (NCCLS) guidelines recommends using Mueller-Hinton agar medium for bacteria and Sabouraud dextrose agar medium for *Candida* [24,25]. Wells were punched in the agar plates (6 mm wide) and inoculated with different bacteria and *Candida*. The wells were filled with 100 μL of the tested compound and the plates were incubated at 37 °C for 24 h. The diameters of the inhibition zones were measured in millimeters (mm). Each antimicrobial assay was performed in triplicates and mean values were reported. Standard antibiotics, gentamicin (10 μg/disc), and nystatin (25 μg/disc) served as positive controls for antimicrobial and *Candida* activity, respectively. 

Solvent control wells of dimethyl sulfoxide (100 μL of DMSO) were used to aid in solubilizing Nystatin and they were used as negative control. The inhibition zone diameters were measured. The organisms used and zone of inhibition to the corresponding compounds are shown in Table 1. 

#### 3.4.3. Serial Dilution Method (Broth Microdilution Assay)

According to the National Committee for Clinical Laboratories Standards (NCCLS), a broth microdilution method was used to determine the minimum inhibitory concentration (MIC) and minimum bactericidal/fungicidal concentration (MBC/MFC) [25,26]. The MIC of a compound was defined as the lowest concentration of the compound that resulted in complete inhibition of visible bacterial/fungal growth at 24 h. The inocula of the bacterial strains or Candida were prepared from overnight broth cultures and suspensions were adjusted to 0.5 McFarland standard turbidity. A serial doubling dilution of the compounds was prepared in a 96/well microtiter plate. A double strength of Mueller Hinton broth/Sabouraud Dextrose broth was used as a diluent. The concentrations were in range of 25–0.02 mg/mL. Bacterial strains and *Candida* were inoculated in Mueller Hinton broth and Sabouraud dextrose broth respectively and inoculated into wells (the final concentration in each well adjusted to 2.0 × 10^6^ CFU/mL for bacteria and 2.0 × 10^5^ of *Candida* strains). The plate was incubated for 24 h at 37 °C. A control well containing the growth medium and the bacteria or *Candida* was set-up. Gentamicin and nystatin served as positive controls, while the solvent (DMSO) was used as a negative control. MIC was defined as the lowest concentration of compound that resulted in complete inhibition of visible (turbidity in the broth) bacterial/fungal growth at 24 h. To determine MBC/MFC broth was taken from each well and inoculated in Mueller Hinton agar for 24 h at 37 °C for bacteria or in Sabouraud dextrose agar for *Candida* strains, respectively. 

The MBC/MFC were defined as the lowest concentration of the compound that kills 99.9% of the original inoculum in 24 h. Tables (1, 2 and 3) show the MIC and MBC/MFC to the corresponding compounds and the organisms used.

#### 3.4.4. Statistical Analyses

Analysis of variance (ANOVA) was used to determine the significance (*p* ≤ 0.05) of the data obtained in all experiments. All results were determined to be within the 95% confidence level for reproducibility. 

### 3.5. Cell Lines and Cell Culture

#### 3.5.1. Materials and Methods

The K562 leukemia cell line was obtained from Dr. Mona Hassona (The University of Jordan, Department of Biology) and was cultured in RPMI; MCF-7 breast cancer cells were obtained from ATCC and were cultured in DMEM. All media were supplemented with 2 mM glutamine and 10% Fetal Bovine Serum (FBS, Gibco Life Technologies) and cells were maintained under standard cell culture conditions at 37 °C in a water-saturated atmosphere of 5% CO_2_ in air.

#### 3.5.2. Cell Proliferation Assay

MCF-7 and K562 cells were seeded at a density of 1 × 10^4^ and 4 × 10^4^ per well in 96-well plates in appropriate medium. For anti-MCF-7 screening, the cells were treated with 50 µM concentrations of the tested compounds. For the IC_50_ determination the cells were treated with increasing concentrations of the tested compound (1.56–100 µM). In all assays, the drugs were dissolved in DMSO immediately before the addition to cell cultures and equal amounts of the solvent were added to control cells. Cell viability was assessed, after 3 days of treatment, with tetrazolium dye 3-(4,5-dimethylthiazol-2-yl)-2,5-diphenyltetrazolium bromide (MTT), obtained from Sigma (Dorset, UK). IC_50_ concentrations were obtained from the dose-response curves using Graph Pad Prism Software 5 (GraphPad Software, Inc. San Diego, CA, USA) [27], and doxorubicin as positive control.
